# Can Digital Technologies Be Useful for Weight Loss in Individuals with Overweight or Obesity? A Systematic Review

**DOI:** 10.3390/healthcare12060670

**Published:** 2024-03-16

**Authors:** Carmela Protano, Andrea De Giorgi, Federica Valeriani, Elisa Mazzeo, Stefano Zanni, Luigi Cofone, Gabriele D’Ancona, Anis Hasnaoui, Ivano Pindinello, Marise Sabato, Francesca Ubaldi, Veronica Volpini, Vincenzo Romano Spica, Matteo Vitali, Francesca Gallè

**Affiliations:** 1 Department of Public Health and Infectious Diseases, Sapienza University of Rome, 00185 Rome, Italy; carmela.protano@uniroma1.it (C.P.); andrea.degiorgi@uniroma1.it (A.D.G.); elisa.mazzeo@uniroma1.it (E.M.); stefano.zanni@uniroma1.it (S.Z.); luigi.cofone@uniroma1.it (L.C.); gabriele.dancona@uniroma1.it (G.D.); ivano.pindinello@uniroma1.it (I.P.); marise.sabato@uniroma1.it (M.S.); matteo.vitali@uniroma1.it (M.V.); 2 Department of Movement, Human, and Health Sciences, University of Rome Foro Italico, 00135 Rome, Italy; f.ubaldi@studenti.uniroma4.it (F.U.); v.volpini@studenti.uniroma4.it (V.V.); vincenzo.romanospica@uniroma4.it (V.R.S.); 3 Faculty of Medicine of Tunis, Tunis El Manar University, Rue Djebal Lakhdar, Tunis 1006, Tunisia; anis.hasnaoui@fmt.utm.tn; 4 Signals and Smart Systems Lab L3S, National Engineering School of Tunis, Tunis El Manar University, Campus Universitaire Farhat Hached, Tunis 1068, Tunisia; 5 Department of Medical, Movement and Wellbeing Sciences, University of Naples Parthenope, 80133 Naples, Italy; francesca.galle@uniparthenope.it

**Keywords:** digital technologies, wearable devices, weight loss, overweight, obesity

## Abstract

Digital technologies have greatly developed and impacted several aspects of life, including health and lifestyle. Activity tracking, mobile applications, and devices may also provide messages and goals to motivate adopting healthy behaviors, namely physical activity and dietary changes. This review aimed to assess the effectiveness of digital resources in supporting behavior changes, and thus influencing weight loss, in people with overweight or obesity. A systematic review was conducted according to the PRISMA guidelines. The protocol was registered in PROSPERO (CRD42023403364). Randomized Controlled Trials published from the database’s inception to 8 November 2023 and focused on digital-based technologies aimed at increasing physical activity for the purpose of weight loss, with or without changes in diet, were considered eligible. In total, 1762 studies were retrieved and 31 met the inclusion criteria. Although they differed in the type of technology used and in their design, two-thirds of the studies reported significantly greater weight loss among electronic device users than controls. Many of these studies reported tailored or specialist-guided interventions. The use of digital technologies may be useful to support weight-loss interventions for people with overweight or obesity. Personalized feedback can increase the effectiveness of new technologies in motivating behavior changes.

## 1. Introduction

Obesity was classified as a disease as early as 1948, and due to the rising epidemic, the World Health Organization (WHO) has since defined obesity as “abnormal or excessive fat accumulation that may impair health”, recognizing the need for action against this epidemic growth [1,2]. In the past two decades, the rates of obesity have rapidly increased across the developing world, and new statistics show that the prevalence of obesity is still growing [3]. It is also estimated that by 2030, obesity will affect over one billion people worldwide [4,5]. The continuous increase in the prevalence of overweight and obesity represents a major public health issue because scientific evidence has demonstrated that these conditions are a risk factor for several diseases, mainly chronic ones, such as diabetes, musculoskeletal disorders, cardiovascular diseases or even some cancers, such as gastroesophageal, breast, endometrial, ovarian, kidney and colon cancer [6,7,8,9]. Since the start of the International Obesity Task Force (IOTF) in 1995 [10], obesity has been calculated based on the body mass index (BMI) which is calculated based on the weight (in kg)/height (in m^2^) ratio [11]. This measurement allows us to classify individuals into the “underweight”, “normal weight”, “overweight”, or “obese” category. The WHO often classifies adult obesity in subclasses [Obese I, II, III] using BMI cutoffs [12]. This WHO classification is beneficial in distinguishing individuals who may have an increased risk of morbidity and mortality due to obesity [2]. Different determinants of health have been associated with obesity, such as individual, socio-economic, lifestyle and environmental factors [13]. It is widely acknowledged that there is a strong correlation between socio-economic status and malnutrition [14]. Some authors state that rapid urbanization can lead to “incorrect food choices” due to high consumption of ultra-processed food. The lack of time and education, in combination with the issue of poverty in this fast-paced world, can lead to poor food choices with a lack of nutritional value and quality and excessive sugar intake, along with a lack of physical activity (PA), which can lead to obesity [15,16]. Different methods for managing weight loss in individuals with overweight or obesity have been developed. These include different types of diets, pharmacotherapy and lifestyle interventions, alone or in combination. However, there is no one-size-fits-all approach, and new strategies are constantly being developed to keep up with changing population trends [17]. Furthermore, notwithstanding their effectiveness in determining weight loss, these methods may be ineffective in long-term body weight maintenance.

The introduction of new technologies has had a huge impact on lifestyle choices and health. In this modern era, in which connectivity and technological innovation are in, smartphones and wearables have rapidly gained popularity. Most of the population have their smartphones on or close to them throughout the day [18,19]. This increase in technology use has also contributed to the increasing adoption of sedentary lifestyle and to the consequent decrease in PA, which can be related to premature mortality and morbidity and an increased risk of major noncommunicable diseases [20]. On the other hand, many researchers have studied different ways to show how the use of digital eHealth or mHealth and new technology, such as wearable sensors, can actually enhance health promotion and prevention [21]. The term mHealth was first invented to describe emerging mobile communications and network technologies for healthcare [22], but later, the WHO defined mHealth as an integral part of eHealth, which refers to the cost-effective and secure use of information and communication technologies in support of health and health-related fields [23]. Good use of mobile phones and related apps can be effective in the delivery of information and improve the impact of treatment and healthcare delivery processes [24]. Likewise, wearable activity trackers such as fitness trackers, activity-tracking smartwatches and pedometers have shown to be very useful tools for overcoming physical inactivity and obesity. Many studies have shown that the use of these devices has been associated with increased PA, since they can support behavior-change techniques like self-monitoring and goal setting, as well as with improved BMI and lower risk of developing obesity [25,26,27,28,29,30]. In 2021, Berry et al. published a systematic review on the effectiveness of digital self-monitoring for weight loss in overweight and obesity, providing positive results in favor of new technologies [31]. In order to add further evidence to this field, the present review was performed to systematically analyze the available literature regarding behavioral weight loss interventions which aimed to increase participants’ PA level by using digital technologies. 

## 2. Materials and Methods

### 2.1. Selection Protocol and Search Strategy

This systematic review was conducted according to the Preferred Reporting Items for Systematic Review and Meta-Analysis (PRISMA) guidelines [32]. The protocol was then registered in PROSPERO with the number CRD42023403364. The research question of the present systematic review was: “Are digital technologies effective to support weight loss in behavioral interventions for individuals with overweight or obesity?”. Thus, the review question was conceived using the “PICOS” Framework (P = Patient, problem or population; I = Intervention; C = Comparison, control or comparator; O = Outcome(s); S = Study type) according to the following eligibility criteria: (P) population: humans with overweight or obesity; (I) intervention: weight loss behavioral intervention based on electronic devices, mobile apps, artificial intelligence or smartphones/watches; (C) comparison: obese and overweight patients who did not undergo weight loss intervention based on electronic devices, mobile apps, artificial intelligence or smartphones/watches; (O) outcome: weight loss, BMI changes, anthropometric measures or body composition; (S) study: clinical trials. After a preliminary assessment of the literature, we decided to restrict the analysis to humans with obesity or overweight without any other comorbidities and to randomized clinical trials in order to obtain more consistent outcomes. Three electronic databases (PubMed, Scopus and Web of Science) were then scrutinized using the following search string: (obesity OR overweight) AND (“artificial intelligence” OR “machine learning” OR “mobile applications” OR “wearable electronic devices” OR smartphone OR smartwatch) AND (“dietary interventions” OR “nutritional status” OR “personalized nutrition” OR “weight control” OR “diet control” OR “weight loss”). Table S1 reports the search strategy for PubMed.

All databases were searched by title, abstract, and MeSH terms and keywords. The last search was performed from database inception to 8 November 2023.

### 2.2. Inclusion and Exclusion Criteria

This review was based on the use of electronic devices and new technologies to increase physical activity with the aim of achieving weight loss. In order to be eligible, studies were selected based on the following inclusion criteria: studies must be in English or Italian; weight loss must be associated with the use of electronic devices, mobile apps, artificial intelligence, or the use of a smartphone/smartwatch to manage/promote physical activity. Only randomized clinical trials were included. Furthermore, all studies which included underage individuals (<18 years) or patients who had other comorbidities or did not present with obesity or overweight were excluded from this systematic review. Reviews, meta-analysis, observational studies, case studies, proceedings, qualitative studies, editorials, commentary studies, pilot studies and any other type of article were also excluded. The references of reviews and meta-analyzes regarding the same issue were checked in order to identify further articles that did not come up on the baseline research results. 

All results, from the beginning until to 8 November 2023, were then retrieved to reference software Zotero Systematic Review Manager v 6.0.26 for further screening and for the removal of duplicates. Ten authors (A.D.G., S.Z., E.M., F.U., V.V., L.C., M.S., G.D.A., I.P., A.H.) then proceeded with the selection of studies by Title and Abstracts according to the selection criteria listed above. All full texts were then read, independently, by the same authors and discussed further. Doubts and disagreements were settled by the other three authors (C.P., F.G., F.V.).

### 2.3. Data Extraction and Quality Assessment

Data were extracted from the selected studies by ten authors (A.D.G., S.Z., E.M., F.U., V.V., L.C., M.S., G.D.A., I.P., A.H.), according to specific characteristics which were previously approved by all authors. The data extraction table was constructed as follows: author, year, country, study design, study population, sample size, type of device, type of intervention, duration, frequency, comparison, main outcomes and secondary outcomes and results. These data were then arranged according to the type of study and the confounding factors. 

Each included article was assessed using the Checklist to Evaluate a Report of a Non-pharmacological Trial (CLEAR NPT) [33]. This checklist has been specifically developed for measuring the quality of randomized clinical trials assessing nonpharmacological treatments. Indeed, the evaluation of nonpharmacological treatments such as technical devices, behavioral or psychological therapy involves some specific methodological considerations. For example, in nonpharmacological treatment trials, it is frequently impossible to carry out the blinding of care providers and participants, and the success of the treatment often depends on the experience and skill of the care providers. Besides, this kind of study is difficult to standardize [33]. Thus, according to several systematic reviews evaluating nonpharmacological treatment [34,35,36,37], the CLEAR NPT checklist was used [33]. This checklist contains 10 parameters, and for each item the choice was between “Yes”, “No” or “Unclear”. By adding up the answers, all authors could attribute a score. The score was between 10 and 8 for a low risk of bias, between 7 and 5 for a median risk of bias and lower than 5 for a high risk of bias. 

The quality assessment was performed independently by ten authors (A.D.G., S.Z., E.M., F.U., V.V., L.C., M.S., G.D.A., I.P., A.H.) and the score was then verified by the other three authors (C.P., F.G., F.V.).

## 3. Results

**Figure 1:**
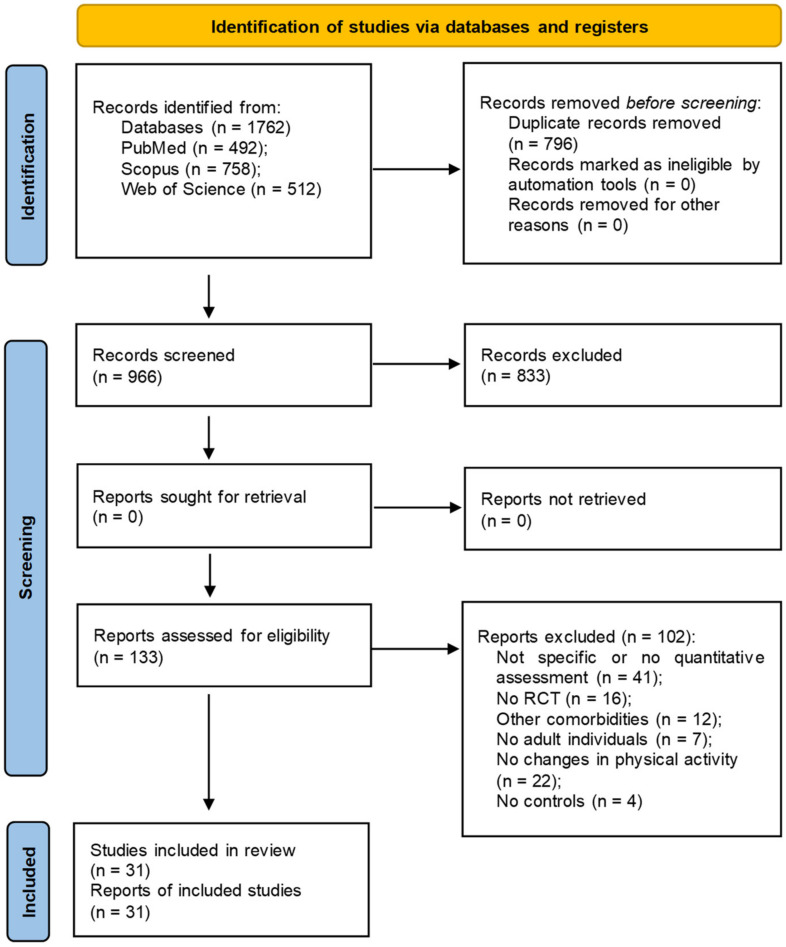
PRISMA flowchart for search strategy.

A total of 1762 studies were retrieved from the following databases: PubMed, Web of Science, and Scopus. Of these, 796 duplicates were removed and 966 were screened by title and abstract. After the full-text assessment of the 133 articles that remained, 102 articles were excluded, 42 of them because they did not pertain to our question, 12 because the individuals were affected by other comorbidities, 16 because they were a different type of study from RCT, 7 because they considered a young age population (<18 years), 4 because did not have control groups, and 21 because they did not consider the assessment of changes in PA. Finally, we included 31 articles that met the inclusion criteria (Figure 1) [38,39,40,41,42,43,44,45,46,47,48,49,50,51,52,53,54,55,56,57,58,59,60,61,62,63,64,65,66,67,68].

The main characteristics and findings of the interventions, as well as the primary and secondary weight-related outcomes assessed alongside weight loss, are shown in Table 1. 

The included articles were published between 2013 [54] and 2023 [61,68], and 15 of them were performed in the USA [40,41,42,45,46,51,54,55,58,62,63,64,65,67], 9 in Europe [38,39,47,49,56,57,59,61,66], 5 in Asia [43,47,52,60,68] and 2 in Australia [44,50]. Both genders were represented in most studies, except in two studies that did not report this information [50,54], and two studies that included only women [47,48]. The overall sample size had a range from 28 [67] to 650 [57]. As for participants’ age, individuals aged 18–80 years were included [48]. All of the studies assessed a BMI mean value with standard deviation, except for that of Hong et al. [48], which reported only the population mean weight. 

In concern to quality assessment, 14 studies were considered with a “Low Bias Risk”, 12 with a “Medium Bias Risk” and 5 with a “High Bias Risk”.

Many of the evaluated studies used smartphone apps to carry out the intervention, matched with other procedures such as motivational phone calls [41] and text messages [50,62], and a good number of them also assessed the use of wearable devices such as smartwatches, smart bands or accelerometers [44,48,51,54,56,57,58,63,67]. 

The majority of the studies included a specific duration of each session and frequency of intervention, with a minimum of 8 weeks [40] and a maximum of 24 months [51] for the duration, and with frequency varying from three times daily [42] to monthly [51], except for a few where these characteristics were kept generic, specifying neither duration nor frequency [47,58,59,64].

All but one [48] of the studies were aimed at achieving weight loss through improvements in both diet and PA. 

In six studies, no activity was assigned to the control group [39,47,49,50,59,61], and in two studies, the control group had the only task of self-monitoring [42,51]. 

As for the results, a weight reduction related to the technologies used was observed in the majority of the studies [39,41,43,45,46,49,52,53,54,56,57,58,59,60,61,64,65,66,67,68]. Additionally, six studies described a reduction in body fat among participants [39,41,57,58,64,67] and in nine papers, a decrease in BMI was also showed beyond weight loss [41,49,52,54,55,56,57,64,66,68]. Moreover, some authors reported waist or hip circumference reductions in the intervention groups [39,41,46,57,58,64,67]. Ten studies reported no significant differences in the outcomes between users and controls [38,40,42,44,47,48,50,55,62,63]. Hernandez et al. reported a decrease in body fat, despite no significant difference in weight loss [47], while the study by Jakicic et al. reported a significantly different weight loss in the favor of standard treatment [51].

## 4. Discussion

The findings of this review suggest that using digital technologies may be useful for supporting interventions aimed at reducing excess weight when employed to modify weight-related behaviors, namely PA and diet. In fact, the majority of the controlled trials analyzed reported significantly better outcomes related to weight loss among participants who used some kind of electronic devices or applications than among non-users [39,41,43,45,46,49,52,53,54,56,57,58,59,60,61,64,65,66,67,68].

The adoption of new technologies is rapidly spreading in several areas of our lives, such as in health promotion and control [69]. In this context, several devices and applications have been proposed as digital solutions to improve health-related behaviors, such as PA and diet, especially since the beginning of the COVID-19 pandemic [70]. As for PA, nowadays, the use of even more sophisticated wearable devices goes beyond the mere tracking of steps or other movements and may help users to reach their activity goals, increase their PA levels and reduce health risk related to inactivity [71]. The integration of gamification and/or social support elements can increase their effectiveness in movement promotion, both in adults and children [72,73,74]. 

With regard to diet monitoring and management, several digital technologies have been developed and evaluated in different subgroups, with inconsistent results [75,76]. Digital resources can reach many people at a low cost and have the potential to support lifestyle changes, enabling individuals to self-regulate their behaviors [77,78,79]. As for employing these technologies for weight loss, a systematic review and meta-analysis published by Berry et al. in 2021 analyzed the potential role of a digital diet and PA self-monitoring in supporting weight loss among adults with overweight or obesity [31]. Their results showed a statistically significant effect of digital self-monitoring in weight loss, moderate PA increase and calorie intake reduction. Furthermore, they reported that tailored interventions were significantly more effective than nontailored ones, highlighting the importance of tailored advice. In line with this, the review by Irvin et al., which was aimed at examining the status of digital exercise program delivery, found that apps may be useful for a low-intensity approach and can improve adherence to programs through self-monitoring [70]. However, the authors stated that tailored interventions can produce significant findings for weight loss and that individuals need specialist support to achieve their weight goals. Interestingly, this has also been proven for digital interventions used in studies aimed at dietary behavior change [80]. Although it was established that digital interventions have the potential to determine proper changes in the eating behavior of individuals, the efficiency of these interventions increases when coupled with tailored feedback and counseling. This should be considered in the perspective of the long-term maintenance of healthy habits after the conclusion of weight loss interventions. 

Keeping this in mind, the evidence coming from our review underlines the usefulness of digital technologies in supporting weight loss, since two-thirds of the analyzed studies showed that their usage resulted in significantly greater weight loss. Furthermore, eighteen of the included studies reported tailored interventions, and only four of these did not find significant differences between participants and controls [42,47,50,63]. In addition, only three [48,50,63] out of the eleven interventions which involved specialists in their implementation reported non-significant differences. The study published by Jakicic et al. was the only reporting that the digital technologies employed for physical activity monitoring and feedback did not offer an advantage over standard behavioral approaches, since the weight reduction observed in its intervention group, although significant, was lower than that observed in controls [51]. Notably, this intervention was not tailored or specialist-driven. 

Digital self-monitoring enables individuals to monitor their health behaviors, either through the input of their own data or through the automatic tracking of sensors or wearable technology. Such solutions can allow individuals to receive tailored, automated and real-time feedback. The integration of these systems into usual weight management services may also inform obesity treatment and address service provision, increasing their effectiveness in weight loss and long-term maintenance [31]. 

However, some considerations are needed in this regard. In general, internal (i.e., motivation and self-efficacy), social (i.e., supporters and saboteurs) and environmental (i.e., an obesogenic environment) factors have been shown to influence the outcomes of a weight loss program, as well as the acceptability of the intervention [81]. Considering the barriers to exercise and PA that people with overweight or obesity may encounter, digital solutions have the potential to provide convenient and equitable support in weight loss based on behavior change [70]. However, as evidence shows that individualized and interactive tools may improve adherence to intervention and facilitate behavior change, those factors which can drive or hinder the use of digital technologies should be also considered when designing a digital-based intervention. In 2022, Jakob et al. reported that user-friendly and technically stable app design, customizable push notifications, personalized app content, passive data tracking, integrated app tutorials, gratuitousness and personal support represent intervention-related characteristics, which can positively influence adherence to mHealth apps for preventing or managing noncommunicable diseases [82]. As for individual-related factors, lack of technical competence, low health literacy, low self-efficacy, a low education level, mental health burden, lack of experience with mHealth apps, privacy concerns, low expectations of the app, low trust in healthcare professionals conducting the intervention, lack of time, age, gender and pre-existing conditions were the user characteristics frequently associated with low mHealth app adherence [82]. 

In addition, due to the availability of different technological solutions, it should also be considered that some of them can be more effective in supporting certain categories than others in behavior change. In a review published in 2018, Cheatham et al. assessed the efficacy of wearable activity tracking technology in assisting behavior change and weight loss, showing that its use in short-term interventions may lead to better results in middle-aged and older adults, but not in younger adults [83]. Belegoli et al. showed that web-based digital health interventions can be more effective in short-term but not in long term weight loss and lifestyle habit changes interventions with respect to offline interventions for overweight and obese adults [84]. 

Therefore, further research in this field should focus on the individualization of digital-based interventions based on subjects’ characteristics. This could imply the choice of the most adequate behavior change technique to motivate people, but also the implementation of educational interventions to increase their digital literacy, and subsequently their adherence to the weight loss program. 

This review has some limitations. First of all, the heterogeneity of the studies examined was high due to the characteristics of the interventions and, in particular, due to the variety of technologies employed and the type of activity (or non-activity) assigned to controls. This did not allow us to compare the studies and to perform a meta-analysis of their results. Furthermore, it should be noted that, in a part of the studies, digital technologies were used to address participants’ dietary behaviors together with PA, while in other interventions, diet was only self-reported or in some cases not controlled at all. This may limit the reliability of the findings related to the effectiveness of each technology in determining a specific behavior change and then weight loss, due to possible confounding bias. Moreover, it should be noted that participants in the studies showed differences in gender, age and health conditions. Although we selected only those studies which involved healthy subjects, it is possible that different categories of subjects, mainly those who perceived themselves as at risk for some disease, complied differently with the intervention and this may have influenced the outcomes. In order to obtain stronger evidence about the effectiveness of technology in weight loss, future research should be focused on specific population subgroups and type of device/application. However, it is also possible to highlight the strengths related to this review. In particular, the analysis was specifically focused on randomized controlled studies involving healthy subjects in order to obtain more reliable evidence. Furthermore, this review was intended to explore the possible employ of digital technology in the context of behavioral interventions aimed at reducing body weight, besides the exclusive use of monitoring devices such as activity trackers.

## 5. Conclusions

As the development of digital technologies advances, their use in healthcare settings increases. Electronic devices and mobile applications may be useful to support weight loss lifestyle-based interventions for people with overweight or obesity. However, evidence suggests that tailored automated feedback or specialists’ advice can increase the effectiveness of these resources by enhancing individuals’ motivation to change their behaviors.

## Figures and Tables

**Table 1:** Characteristics of the included studies.

Author Year Country	Sample Size Study Population	Type of Device	Type of Intervention, Duration, Frequency	Comparison	Main and Secondary Outcomes	Results	Quality
Apinaniz et al., 2019 Spain [38]	110, 54 intervention and 56 controls; 38.5 ± 5 years; 72% F and 28% M; BMI 32.7 ± 4.9 kg/m^2^	Smartphone app AKTIDIET	The AKTIDIET app on patients’ smartphones provided reinforced health advice, including exercise programs, food intake tracking and instructional videos. Patients followed up at 1, 3 and 6 months. The program required daily self-reporting of diet and exercise, with personalized feedback and weekly assignments. The examination was repeated at 16 and 38 weeks	Usual care and motivational advice including recommendations on diet and physical exercise	Body weight after 6 months and adherence to dietary and exercise recommendations	There were no significant differences in weight change, nor in the adherence to dietary or physical exercise recommendations	6 Yes, 3 No, 1 Unclear; Medium Bias Risk
Balk-Møller et al., 2017 Denmark [39]	566, 355 intervention and 211 controls; 47 ± 10 years; 92.2% F and 7.8% M; BMI 73.8 ± 15.4 kg/m^2^	Web and smartphone app SoSu-life	Daily self-reporting of diet and exercise, personalized feedback about specific health issues related to the chosen pledge, with weekly assignments and challenges. The examination was repeated at 16 and 38 weeks	No activities	Change in body weight and anthropometric markers	The SoSu-life group had a larger decrease in body weight (−1.01 kg, *p* = 0.03), body fat percentage (−0.78%, *p* = 0.03), and WC (−1.79 cm, *p* = 0.007) after 38 weeks compared with the control group. The SoSu-life group had a larger decrease in body weight (−1.54 kg, *p* < 0.001) and a decrease in body fat percentage of −0.81% (*p* = 0.003) compared with the control group during the first 16 weeks	9 Yes, 0 No, 1 Unclear: Low Bias Risk
Beatty et al., 2020 USA [40]	72, 37 intervention and 35 controls; 37.7 ± 15.3 years, 65.3% F and 34.7% M; BMI 31.3 ± 3.2 kg/m^2^	A wrist-worn ELMM device capable of tracking bites, displayed after each meal, as well as the number of steps taken by the user.	WD for 8 weeks. Workbook offered education regarding eating rate, energy intake and energy expenditure.	WO	Weight loss	No significant difference between WD and WO groups with respect to weight change [−0.46 (1.11) vs. 0.26 (0.82) kg, respectively, *p* = 0.40]	4 Yes, 2 No, 4 Unclear; High Bias Risk
Block et al., 2015 USA [41]	339, 163 intervention and 176 controls; 55 ± 8.9 years; 31.3% F and 68.7%; BMI 31.2 ± 4.4 kg/m^2^	Alive-PD program via Web, smartphone and automated phone calls	The program offered personalized dietary and PA goals, tracking tools, health information, quizzes, social support, feedback and reminders via web, email, IVR phone calls and mobile. The program lasted for a year, with regular goal setting and contact. Users received goals weekly for the first six months and bi-weekly thereafter, plus midweek reminders.	No contact from Alive-PD system except reminders to complete a 3-month and 6-month online follow-up questionnaire	Changes in body weight, BMI, WC	Reductions in weight, BMI and WC were all significantly greater in the intervention group than the control group (*p* = 0.01)	9 Yes, 0 No, 1 Unclear; Low Bias Risk
Burke et al., 2022 USA [42]	502, 251 intervention and 251 controls; 45.0 ± 14.4 years; 79.5% F and 20.5% M; BMI 33.7 ± 4.0 kg/m^2^	Fitbit Charge 2, smartphone app	SM+FB of diet, PA and weight in a behavioral weight-loss intervention at 6 and 12 months. The calorie goal was determined based on the person’s baseline body weight and real-time synced SM data to send messages that were responsive to the participants’ SM entries; wrist-worn Fitbit Charge 2 was used to self-monitor PA with an aim of 150 min/week by 12 weeks. Participants weighed themselves daily. In-app messages were sent 3 times daily over the 12-month intervention	SM	Weight loss and changes in BMI from baseline to 6 months, percentage of body fat, WC	At 6 months, there was a significant percentage of weight change in both groups (SM+FB: −3.16%, 95% CI: −3.85% to −2.47%, *p* < 0.0001; SM: −3.20%, 95% CI: −3.86% to −2.54%, *p* < 0.0001) but no significant between-group mean difference (−0.04%, 95% CI: −0.99% to 0.91%, *p* = 0.940).	9 Yes, 0 No, 1 Unclear; Low Bias Risk
Cho et al., 2020 Republic of Korea [43]	129, 88 intervention and 41 controls; 49.2 ± 7.7 years; 51.2% F and 48.8% M; BMI 26.3 ± 3 kg/m^2^	Smartphone app	An app-based diet and exercise self-logging group (app only), or app-based self-logging and personalized coaching from professional dieticians and exercise coordinators group. The app delivered structured health-related curricula and personalized feedback based on reviews of the user’s logs. Assessments were performed at baseline, week 6, week 12 and week 24	Baseline education; no apps	Weight changes, body fat mass, WC between baseline and follow-up assessments	Those using the app with the personalized coaching group had greater body weight reductions (control −0.12 ± 0.30 kg; app only −0.35 ± 0.36 kg, *p* = 0.67; app with personalized coaching −0.96 ± 0.37 kg, *p* = 0.08), specifically by body fat mass reduction (control −0.13 ± 0.34 kg; app only −0.64 ± 0.38 kg, *p* = 0.22; app with personalized coaching −0.79 ± 0.38 kg, *p* = 0.08)	9 Yes, 0 No, 1 Unclear; Low Bias Risk
Duncan et al., 2020 Australia [44]	116, 39 Enhanced, 41 Traditional and 36 Control; 44.5 ± 10.4 years; 70.7% F and 29.3% M; BMI 31.7 ± 3.9 kg/m^2^	Smartphone app Balanced, Fitbit, Accelerometer (Geneactiv)	In a 6-month intervention, Enhanced and Traditional group participants received personalized dietary recommendations, access to the ‘Balanced’ smartphone app, a calorie-counting platform, a face-to-face dietary consultation, a Fitbit activity tracker, body weight scales and a handbook.	The waitlist control group was asked to maintain current weight, PA and dietary intake	Weight change	At 6 months, weight was not significantly different between the pooled intervention groups and control group (difference = −0.92, 95% CI (−3.33, 1.48)) or 12 months (difference = 0.00, 95% CI (−2.62, 2.62)).	8 Yes, 2 No, 0 Unclear; Low Bias Risk
Farage et al., 2021 USA [45]	191, 103 intervention and 88 controls; 34.8 ± 7.6 years; 51.8% F and 48.2% M; BMI 46% 25–30 and 54% over 30 kg/m^2^	Smartphone app Lose it!	Electronic diet and exercise self-monitoring and weight loss interventions on 4- and 12-month weight loss; 28 phone calls over 12 months with counselors, regular feedback through email and weight monitoring using the BodyTrace e-scale. In addition, the participants received a personalized exercise plan based on their self-reported baseline PA. They were asked to gradually increase aerobic exercise from their baseline level until reaching 225–250 min weekly	Self-paced participants received assistance upon request	Weight change	At 4 months, the counselor-initiated treatment group lost an average of 3.7 kg (SD 3.6), and the self-paced treatment group lost 0.6 kg (SD 3.1). At 12 months, the counselor-initiated treatment lost 2.4 kg (SD 5.0) on average and the self-paced treatment group gained 0.2 kg (SD 5.1).	7 Yes, 2 No, 1 Unclear; Medium Bias Risk
Fukuoka et al., 2015 USA [46]	61, 30 intervention and 31 controls; 55.2 ± 9.0 years; 77% F and 23% M; BMI 33.3 ± 6.0 kg/m^2^	Smartphone app and Omron pedometer	The intervention lasted 5 months and consisted of six in-person sessions and a home-based exercise program. A study-developed mobile phone app and pedometer augmented the intervention and providing self-monitoring tools (recording weight, activity and caloric intake). It was also used to deliver interactive intervention content through daily messages, video clips and quizzes	The control group used the pedometer, but the settings were changed to display the number of steps. No specific step goals were provided. Research staff removed the run-in mobile app from the participant’s iPhone or collected the iPhone if one had been provided	Percentage change in weight and BMI from baseline to 5-month follow-up, hip circumference, objectively measured (via pedometer) PA	The intervention group (n = 30) lost an average of 6.2 (5.9) kg (−6.8% [5.7%]) between baseline and 5-month follow-up compared to the control group’s (n = 31) gain of 0.3 (3.0) kg (0.3% [5.7%]) (*p* < 0.001). The intervention group had greater reductions in hip circumference (*p* < 0.001)	6 yes, 2 no, 2 unclear; Medium Bias Risk
Hernández-Reyes et al., 2020 Spain [47]	90, 45 intervention and 45 controls; 41.5 ± 11.3 years; 100% F; BMI 31.8 ± 5.3 kg/m^2^	Automatic push notifications	Objectives for diet and PA through exclusive access to specific functionalities of the app and automatic push notifications on specific days with personalized health-related and motivational messages	No access to functionalities related to the self-monitoring of weight at home, gamification or prescription of PA	Body fat loss, muscle mass and weight loss at 6 months	Receiving notifications during the intervention increased body fat loss (mean −12.9% [SD 6.7] in the intervention group vs. mean −7.0% [SD 5.7] in the control group; *p* < 0.001) and helped to maintain muscle mass (mean −0.8% [SD 4.5] in the intervention group vs. mean −3.2% [SD 2.8] in the control group; *p* < 0.018). These variations between groups led to a non-significant difference in weight loss (mean −7.9 kg [SD 3.9] in the intervention group vs. mean −7.1 kg [SD 3.4] in the control group; *p* > 0.05).	4 Yes, 3 No, 3 Unclear; High Bias Risk
Hong et al., 2022 Republic of Korea [48]	29, 12 intervention and 17 controls; 80 ± 3.3 years; 100% F; Weight 58.63 ± 8.17 kg	Smartphone, 24-inch LCD display monitor and a smartphone mirroring device (Miracast MRC-01, Actto)	Smartphone mirroring-based telepresence exercise Program with exercise instructor who had a major in exercise physiology, in which participants exercised in their homes for 20–40 min three times a week for 12 weeks. Nutrition advice and fitness monitoring once a month.	Same exercise program at the senior citizen center	Weight loss, body composition and physical function	Weight (*p* = 0.006) significantly decreased in the control group, body fat percentage decreased significantly in the intervention (*p* = 0.026) and in the control (*p* = 0.001) groups, and skeletal muscle mass (*p* = 0.44) significantly increased in the control group. Two-way repeated-measures ANOVA revealed no significant interaction effects on all variables.	8 Yes, 0 No, 2 Unclear; Low Bias Risk
Hurkmans et al., 2018 Belgium [49]	102, 80 intervention and 22 controls; 45.5 ± 10.3 years; 70% F and 30% M; BMI 32 ± 2.0 kg/m^2^	Smartphone app	All intervention groups received the same advice from a registered dietician and a qualified PA coach during a 12-week period. The methods used included a conventional face-to-face weight loss program, a weight loss app program (app group) and a partial face-to-face and partial app program (combi group)	The control group did not receive any information during the same period	Weight reduction (5% decrease), BMI, metabolic risk factors, dietary pattern and PA	In the conventional group, app group, and combi group, BMI decreased significantly (*p* = 0.004, *p* = 0.005, and *p* < 0.001, respectively), no significant decrease was found in the control group. A significant time x group effect was found for BMI (*p* = 0.006), with the control group being significantly different compared with all other intervention groups. No significant differences were found between the conventional group and the app group and between the conventional group and the combi group (*p* = 0.41). However, the combi group had significantly higher decrease in BMI compared with the app group (*p* = 0.03).	8 Yes, 0 No, 2 Unclear; Low Bias Risk
Hutchesson et al., 2018 Australia [50]	57, 29 intervention and 28 controls; 27.1 ± 4.7 years; unspecified gender; BMI 29.4 ± 2.5 kg/m^2^	Advice via smartphone app, SMS, emails and website	Six-month weight loss program delivered using e-Health technologies only, comprising five delivery modes (website, app, email, text messages and social media) and using social cognitive theory and control theory theoretical frameworks. Participants received automated personalized email feedback from their accredited practicing dietitian. Individualized energy intake and energy expenditure goals were set for each participant based on their estimated energy expenditure and creating a 2500 kJ/day energy deficit to help facilitate a 0.5–1 kg weight loss/week, goals to be achieved by modifying eating and physical habits	No intervention for six months: they were instructed to continue their usual eating and PA habits	Weight change at six months	No significant between-group differences were observed for weight (*p* > 0.05); significant mean difference favoring the intervention group was observed for body fat (kg) (−3.10 (−5.69, 0.52), *p* = 0.019).	8 Yes, 0 No, 2 Unclear; Low Bias Risk
Jakicic et al., 2016 USA [51]	471, 237 intervention and 234 controls; mean 30.9 years; 71% F and 29% M; mean BMI 31.2 kg/m^2^	Wearable device and web interface	Low-calorie diet, PA, and group counseling sessions. At 6 months, telephone counseling sessions and text message prompts were added to the interventions, with self-monitoring of diet and PA using a website (standard intervention) or a wearable device (enhanced intervention). Group-based sessions were scheduled weekly for the initial 6 months and monthly between months 7 and 24.	Same intervention but only with self-monitoring; no website or wearable devices	Weight change, body composition	At 24 months, weight loss was 2.4 kg (95% CI, 1.0 to 3.7) lower in the enhanced intervention group compared with the standard intervention group (*p* = 0.002). In post hoc analysis, the percent weight loss differed significantly between the standard intervention and enhanced intervention groups (*p* < 0.001). Both groups had significant improvements in body composition, with no significant difference between groups.	9 Yes, 0 No, 1 Unclear; Low Bias Risk
Jiang et al., 2021 China [52]	272, 136 intervention and 136 controls; 31.8 ± 5 years; 41.2% F and 58.8% M; BMI 32.5 ± 3.5 kg/m^2^	Smartphone app and daily online instructions	Six-month intervention. Companion-Intensive Multi-aspect Weight Management (CIMWM) strategy focusing on a combination of online and offline medical interventions with daily lifestyle supervision and guidance of diet and exercise. Participants received an individualized calorie-restricted diet which was developed by registered dietitians. Individualized exercise plans were created by health managers for each participant based on their health status and exercise capacity. Participants in the CIMWM group were provided with two Fit Nutrition Bars daily as well as monthly face-to-face guidance and daily online instructions via the mobile application “Medical Weight Management”, which allowed them to upload data regarding their daily weight, as well as food diaries, lifestyle supervision and guidance of diet and exercise.	Traditional multi-aspect weight management was required to complete daily self-monitoring instead of being offered as daily online instructions	Clinically significant weight loss (defined as weight loss ≥ 5%), anthropometric measures and determination of metabolic indexes	Significant changes in BMI, body fat and skeletal muscle mass-to-visceral fat area ratio from baseline to 6 months were observed between two groups (*p* < 0.05).	5 Yes, 2 No, 3 Unclear; Medium Bias Risk
Johnson et al., 2019 USA [53]	30, 20 intervention and 10 controls; 43.2 ± 11 years; BMI 36.1 ± 6.8 kg/m^2^	Wireless watches and weight scales to sync with personal smartphones	Participants assigned to the VCIP group received individualized health coaching by a multidisciplinary team (registered dietitian, exercise physiologist, certified athletic trainer and medical doctor) based on data uploaded over the 12-week intervention period	m-health devices; no health coaching sessions, nor team member feedback on steps per day nor calories uploaded	Weight change	There was a significant (*p* < 0.001) difference for post-intervention weight loss between VC (8.23 kg) compared to IP (3.2 kg) and CG (2.9 kg)	7 Yes, 1 No, 2 Unclear; Medium Bias Risk
Johnston et al., 2013 USA [54]	292, 147 intervention and 145 controls; 46.5 ± 10.5 years; 90% F and 10% M; BMI 33 ± 3.6 kg/m^2^	WW smartphone application and WW online tools	WW program based on food and activity plan, group support and skills to change behavior, followed through weekly meetings. Weights and self-reported use of access modes were measured at baseline and at 3 and 6 months	Self-help group with publicly available printed materials explaining basic dietary and exercise guidelines for safe weight loss	Reductions in BMI and weight	WW subjects lost 4.6 kg and self-help subjects lost 0.6 kg at 6 months. Participants in the WW group significantly decreased their weight (F = 34.5, *p* < 0.001) and BMI at 6 months (F = 36.7, *p* < 0.001) compared with those in the self-help group	6 Yes, 3 No, 1 Unclear; Medium Bias Risk
Laing et al., 2014 USA [55]	212, 105 intervention and 107 controls; 43.1 ± 14.5 years; 73.1% F and 26.9% M; BMI 33.4 ± 7.09 kg/m^2^	Smartphone app	6 months of usual care without (n = 107) or with (n = 105) MyFitnessPal; dietary intake, PA and weight self-monitoring, goal setting, and feedback	Control group patients were free to “choose any activities you’d like to lose weight,” without specifying any particular interventions	Weight loss at 6 months, 3 self-reported behavioral mediators of weight loss (exercise, diet and self-efficacy in weight loss) at baseline and at 3 and 6 months	At 3 months, participants in the control group gained an average of 0.24 kg, whereas those in the intervention group lost 0.03 kg (between-group difference 0.27 kg [95% CI, 1.13 to 0.60 kg]; *p* = 0.53). At 6 months, participants in the control group gained an average of 0.27 kg and those in the intervention group lost 0.03 kg (between-group difference 0.30 kg [CI, 1.50 to 0.95 kg]; *p* = 0.63)	7 Yes, 1 No, 2 Unclear; Medium Bias Risk
Lugones-Sanchez et al., 2020 Spain [56]	440, 231 intervention and 209 controls; 48.1 ± 10 years; 69.3% F and 30.7% M; BMI 32.8 ± 3.4 kg/m^2^	Smartphone app (EVIDENT 3 APP) and Smart band (Mi Band 2, Xiaomi)	3-month intervention with counseling, smartphone app and smart band (Mi Band 2, Xiaomi). After 7 days, subjects were trained to use the device and the app to allow the dietary intake to be self-reported daily and PA data were collected automatically from the smart band. Once all of the daily information was collected, the app integrated the data to create personalized recommendations based on the subjects’ characteristics and specific objectives and goals for weight loss.	Controls only had counseling	Weight loss and changes in some parameters of body composition at baseline and 3 months	The mHealth intervention produced a greater loss of body weight (−1.97 kg, 95% CI −2.39 to −1.54) relative to standard counseling at 3 months (−1.13 kg, 95% CI −1.56 to −0.69): *p* < 0.01. A significant between-group difference was noted only in BMI (−0.54 kg/m^2^, 95% CI −0.84 to –0.24); *p* < 0.01.	8 Yes, 1 No, 1 Unclear; Low Risk of Bias
Lugones-Sanchez et al., 2022 Spain [57]	650, 318 intervention and 332 controls; 48.3 ± 9.6 years; 68.5% F and 31.5% M;BMI 33.04 ± 3.5 kg/m^2^	Smartphone app, wristband, brief counseling	The intervention group received training to use the app and the smart band for 3 months; self-monitoring, tailored feedback and a PA record. The app integrated the data to create personalized healthy food recommendations. The smart band was set to congratulate the user when reaching 10,000 steps/day, and the app displayed this step recommendation	Brief counseling	Weight loss, body composition	At 12 months, significant mean differences were found between groups for weight −0.26 (−1.21 to 0.70), BMI −0.06 (−0.41 to 0.28), waist circumference −0.48 (−1.62 to 0.66), hip circumference −0.69 (−1.62 to 0.25) and body adiposity index −0.33 (−0.77 to 0.11)	8 Yes, 1 No,1 Unclear; Low Bias Risk
Martin et al., 2015 USA [58]	40, 20 intervention and 20 controls; 44.4 ± 11.8 years; 82.5% F and 17.5% M; BMI 29.8 ± 2.9 kg/m^2^	Smartphone app Smartloss and accelerometer	SmartLoss participants (n = 20) were prescribed a 1200 to 1400 kcal/d diet and were provided with a smartphone, body weight scale and accelerometer that wirelessly transmitted body weight and step data to a website. Participants received feedback and treatment recommendations once a week based on their weight graph, while counselors educated each participant that the weight graph was used to objectively quantify adherence to the calorie prescription and to guide counseling and treatment recommendations	Attention-matched health education with health tips on smartphone	Change in body weight and waist circumference	Weight loss was significantly larger in the SmartLoss (least squares mean ± SEM: −9.4 ± 0.5%) compared with the Health Education group (−0.6 ± 0.5%), *p* < 0.001; Mean ± SEM waist circumference change for the SmartLoss group was 21.6 ± 1.00, 25.3 ± 1.01, and 26.9 ± 1.00 cm while in the Health Education group was 1.3 ± 1.04, 1.7 ± 1.04, and 1.7 ± 1.00 cm at weeks 4, 8, and 12, respectively, *p* < 0.05.	6 Yes, 1 No, 3 Unclear; Medium Bias Risk
Martínez-Rodríguez et al., 2022 Spain [59]	80, 40 intervention and 40 controls; 45.7 ± 8.5 years; BMI 32.9 ± 5.1 kg/m^2^	Smartphone app	Dietary and activity recommendations provided with a wearable device (Fitbit Charge 2) and the dietary supplement Metabolaid^®^+ an activity bracelet for monitoring+ smartphone app	Dietary and activity recommendations provided with Fitbit Charge 2 and the dietary supplement + an activity bracelet for monitoring	Weight loss, body composition, anthropometric measurements	Both groups lost a significant amount of body weight (*p* < 0.001), while the group using the app also lowered their fat mass (*p* < 0.005).	3 Yes, 4 No, 3 Unclear; High Bias Risk
Nakata et al., 2022 Japan [60]	141, 72 intervention and 69 controls; 43.2 ± 9.3 years; 26% F and 74% M; BMI 27.6 ± 3.5 kg/m^2^	Smartphone app Healthcare, CALO mama Plus	3-month intervention. Smartphone healthcare application CALO mama Plus registered daily diet, exercise, calculated dietary intake and provided advice using artificial intelligence technology. The participants wore the device for at least 10 h/day for more than 3 days	No intervention; they continued their current lifestyle without any dietary apps	Body weight change over 3 months	The change in body weight was −2.4 ± 4.0 kg and −0.7 ± 3.3 kg in the intervention and control groups, respectively, with a significant between-group difference in body weight change (−1.60 kg; 95% confidence interval −2.83 to −0.38; *p* = 0.011).	6 Yes, 3 No, 1 Unclear; Medium Bias Risk
Roth et al., 2023 Finland [61]	150, 77 intervention and 73 controls; 43.4 ± 10.9 years; 91.3% F and 8.7% M; BMI 35.8 ± 3.2 kg/m^2^	Smartphone app	12 months of healthy lifestyle that supported sustainable weight loss through physical therapy and proper nutrition	No app or electronic devices	Weight loss and changes from baseline to 12 months in body fat distribution	The intervention group lost, on average, 7.75% (95% CI: 9.66% to 5.84%) of their initial body weight after 12 months, whereas the weight of the controls did not change (mean = 0.00% [95% CI: 1.98% to 1.99%]); *p* < 0.001.	6 Yes, 3 No, 1 Unclear; Medium Bias Risk
Saldivar et al., 2021 USA [62]	371, 185 intervention and 186 controls; 54.1 ± 10.5 years; 82.8% F and 17.2% M; BMI 43.1 ± 9.53 kg/m^2^	Smartphone app and text messages	12-week and 20-week texting program—POWER Program with three text messages per week, which included appointment reminders, health and wellness tips and educational information related to care and disease management. The 12- and 20-week programs allowed patients to set goals around exercise or nutrition. The 20-week program also included motivational, mental health and stress management messages to help encourage healthy lifestyle changes	Only medical group visits without any text messages	Weight loss	Both POWER and POWER + 20-week texting groups had a significant reduction in weight at their final group visit compared to their baseline (POWER, 114 ± 27 kg vs. 112 ± 26 kg, *p* < 0.001; POWER + 20-week texting, 111 ± 28 kg vs. 109 ± 28 kg, *p* < 0.01), but not the 12-week texting group (114 ± 29 kg vs. 113 ± 29 kg, *p* = 0.22), with no differences between the groups.	3 Yes, 2 No, 5 Unclear; High Bias Risk
Spring et al., 2017 USA [63]	96, 32 Standard, 32 Technology supported, 32 Self-guided; 39.3 ± 11.7 years, 84.4% F and 15.6% M; BMI 34.6 ± 3.0 kg/m^2^	Smartphone app ENGAGED and wireless accelerometer	6 month intervention. STND and TECH groups received eight 90 min in-person weekly group sessions. TECH used a smartphone application with social networking features and wireless accelerometer, and received 2 to 4 personalized messages per week by trained coaches with at least a bachelor’s degree who reviewed the self-monitoring and goal attainment and helped participants solve problems. If fidelity fell below 90%, the coach was retrained by a doctoral-level staff member	Self-guided (SELF) and Standard (STND) used paper diaries to self-monitor diet, activity and weight	Primary weight loss and behavioral adherence	Weight loss was greater for TECH and STND than SELF at 6 months (25.7 kg [95% confidence interval: 27.2 to 24.1] vs. 22.7 kg [95% confidence interval: 25.1 to 20.3], *p* < 0.05) but not at 12 months. TECH and STND did not differ except that more STND (59%) than TECH (34%) achieved 5% weight loss at 6 months (*p* < 0.05).	9 Yes, 1 No, 0 Unclear; Low Bias Risk
Stephens et al., 2017 USA [64]	62, 31 intervention and 31 controls; median 20 years; 71% F and 29% M; BMI 28.5 kg/m^2^	Smartphone app	Smartphone application + health coach intervention and counseling sessions, providing health coach with the ability to monitor and track all participant progress on a real-time basis and text messages focused on current diet or PA status. Participants were encouraged to exercise at least 150 min/week at moderate intensity	Counseling session	Weight, BMI, WC, dietary habits, PA habits and self-efficacy for healthy eating and PA at 3 months	The control group gained a slight amount of weight (0.3 kg) from baseline to 3 months, while participants in the Smartphone + Health Coach group lost a significant amount (−1.8 kg, *p* < 0.01); the difference in weight change between groups was statistically significant (*p* = 0.026). The smartphone group also had a significant decrease in BMI (*p* < 0.01) and WC (*p* < 0.01)	3 Yes, 2 No, 5 Unclear; High Bias Risk
Thomas et al., 2020 USA [65]	146, 72 intervention and 74 controls; 58.3 ± 10.3 years; 78.1% F and 21.9% M; BMI 91.4 ± 15.6 kg/m^2^	Website and smartphone app	6 months of no-cost access to the online web-based virtual reality program, accessible via website and mobile app. Half of the participants were randomized to also receive the ES) program, which consisted of four separate ‘scenarios’ focused on challenges at home, the workplace, the gym and social gatherings that were made available to participants at weeks 2, 4, 6 and 8, respectively, with daily points goals personalized according to sex, age, starting weight and activity level	Online weight management program alone (WW)	Body weight loss, satisfaction with the weight-loss program	Both groups achieved statistically significant weight loss across the trial, with no difference in mean ± standard error weight loss between WW and WW + ES at 3 months (2.7 ± 1.1 kg vs. 4.2 ± 1.1 kg, respectively; *p* = 0.086) but greater weight loss in WW + ES at 6 months (2.6 ± 1.3 kg vs. 4.9 ± 1.3 kg, respectively; *p* = 0.042)	8 Yes, 0 No, 2 Unclear; Low Bias Risk
Thorgeirsson et al., 2022 Iceland [66]	146, 95 intervention and 51 controls; 46.8 ± 11.7 years; 92.5% F and 7.5%; BMI 36.3 ± 5.2 kg/m^2^	Smartphone app Sidekick	Standard treatment supplemented with a digital therapeutic mobile application designed to increase frequency of healthy behaviors through goal-setting, self-monitoring and completion of health-related tasks in nutrition, PA and stress management for 4 months	Standard weekly coaching sessions for 4 months	BMI at 4 months	The weight loss was 3.6% among those treated per-protocol (n = 70), and 1.5% among those not treated per-protocol (n = 76) (*p* < 0.0001). BMI reductions of 1.4 kg/m^2^ (treated per-protocol) and 0.5 kg/m^2^ (not treated per-protocol) (*p* < 0.0001) were achieved.	5 Yes, 3 No, 2 Unclear; Medium Bias Risk
Vaz et al., 2021 USA [67]	28, 13 intervention and 15 controls; 43.25 ± 2.48 years; 86% F and 14% M; BMI 34.40 ± 0.96 kg/m^2^	A wrist-worn three-axis accelerometer (Fitbit Charge Heart Rate™), a smartscale (Fitbit Aria™) smartphone app Fitbit™ and commercially available messaging and photo-sharing apps	Participants were instructed to step on the smartscale every morning. The app was programmed to automatically send out a reminder to motivate participants to meet the target for PA for that day, based on continuous activity data obtained from the wearable activity tracker, with remote professional coaching by the physician. Participants were instructed to wear the activity tracker as close as possible to 24 h per day, 7 days per week, and any day with <500 recorded steps indicated a tracking problem. They received conventional outpatient weight-management visits every 3 months for 6 months of the duration of the intervention	Controls received only weight-management visits	Change in weight at 6 months, changes in waist circumference	At 6 months, the intervention group experienced a statistically significant weight change of −7.16 ± 1.78 kg (mean ± SE, 95% CI −11.05 to −3.26, *p* < 0.01), which differed from the weight change in controls (−3.00 ± 1.05 kg (95% CI −5.27 to −0.73, *p* < 0.05) by −4.16 ± 2.01 kg (95% CI −8.29 to −0.02, *p* < 0.05). Waist circumference significantly improved (intervention vs. control: *p* < 0.01).	6 yes, 2 no, 2 unclear; Medium Bias Risk
Zhang et al., 2023 China [68]	642, 440 intervention and 202 controls; 46.1% F and 53.9% M; 70.1 ± 5.3 years; BMI 27.67 ± 2.63 kg/m^2^	Smartphone app	The remote dietary and PA intervention group (group DPI), and the remote PA intervention group (group PI) used the app for health information collection, health assessment, guidance and feedback and follow-up. The treatment duration was 3 months. Nutritional professionals provided one-on-one personalized dietary guidance and feedback to the participants according to their age, gender, weight, food intake, chronic disease situation, choice of food type, and portion size, 3 to 5 times a week	Health education book on a reasonable diet	Weight at day 45 (time 2), and day 90 (time 3)	Compared with groups PI and controls, group DPI showed a significant decrease in weight (−1.56 vs. −0.86 kg and −1.56 vs. −0.66 kg, respectively; *p* < 0.05) and BMI (−0.61 vs. −0.33 kg/m^2^ and −0.61 vs. −0.27 kg/m^2^, respectively; *p* < 0.05) at time 2. Compared with groups PI and controls, group DPI showed a significant decrease in body weight (−4.11 vs. −1.01 kg and −4.11 vs. −0.83 kg, respectively; *p* < 0.05) and BMI (−1.61 vs. −0.40 kg/m^2^ and −1.61 vs. −0.33 kg/m^2^, respectively; *p* < 0.05) at time 3.	9 Yes, 0 No, 1 Unclear; Low Bias Risk

Eat Less, Move More (ELMM); workbook plus device (WD); workbook only (WO); self-monitoring (SM); feedback (FB); videoconferencing (VC); in-presence (IP); Weight Watchers (WW); MyFitnessPal app (MyFitnessPal); preventing obesity with eating right (POWER); standard (STND); technology-supported (TECH); experience success (ES); Automated Interactive Voice Response (IVR); body mass index (BMI); confidence interval (CI); physical activity (PA); standard deviation (SD); waist circumference (WC).

## Data Availability

Raw data will be made available, if necessary, upon request to the corresponding author.

## Supplementary Materials

The following supporting information can be downloaded at: https://www.mdpi.com/article/10.3390/healthcare12060670/s1, Table S1 reports the search strategy used for PubMed.
