# Supplementary material for: Can Digital Technologies Be Useful for Weight Loss in Individuals with Overweight or Obesity? A Systematic Review
Source: Healthcare (Basel). 2024 Mar 16;12(6):670. doi: 10.3390/healthcare12060670 (PMC10970199; doi:10.3390/healthcare12060670)
Supplement: Supplementary file 1 [file healthcare-12-00670-s001.zip › healthcare-2864285-supplementary.pdf]

Supplementary Material S1

Table S1 Search strategy

Primary question: Are digital technologies effective to support weight loss in behavioural interventions for individuals with overweight or obesity?

**Draft search strategy for electronic database queried: PubMed**
For each search, no start date was applied, and database was searched from their inception or date of the earliest available publication

<table>
<tr><td>Database</td><td>PubMed 492 results</td></tr>
<tr><td>Description</td><td>Search performed considering terms by “(obesity OR overweight) AND ("artificial intelligence" OR "machine learning" OR "mobile applications" OR "wearable electronic devices" OR smartphone OR smartwatch) AND ("dietary interventions" OR "nutritional status" OR "personalized nutrition" OR "weight control" OR "diet control" OR "weight loss")” and terms controlled by Medical Subjects Headings (MeSH)</td></tr>
<tr><td colspan="2">("obeses"[All Fields] OR "obesity"[MeSH Terms] OR "obesity"[All Fields] OR "obese"[All Fields] OR "obesities"[All Fields] OR "obesity s"[All Fields] OR ("overweight"[MeSH Terms] OR "overweight"[All Fields] OR "overweighted"[All Fields] OR "overweightness"[All Fields] OR "overweights"[All Fields])) AND ("artificial intelligence"[All Fields] OR "machine learning"[All Fields] OR "mobile applications"[All Fields] OR "wearable electronic devices"[All Fields] OR ("smartphone"[MeSH Terms] OR "smartphone"[All Fields] OR "smartphones"[All Fields] OR "smartphone s"[All Fields]) OR ("smartwatch"[All Fields] OR "smartwatches"[All Fields])) AND ("dietary interventions"[All Fields] OR "nutritional status"[All Fields] OR "personalized nutrition"[All Fields] OR "weight control"[All Fields] OR "diet control"[All Fields] OR "weight loss"[All Fields])</td></tr>
<tr><td rowspan="4">Translations</td><td>obesity: "obeses"[All Fields] OR "obesity"[MeSH Terms] OR "obesity"[All Fields] OR "obese"[All Fields] OR "obesities"[All Fields] OR "obesity's"[All Fields]</td></tr>
<tr><td>overweight: "overweight"[MeSH Terms] OR "overweight"[All Fields] OR "overweighted"[All Fields] OR "overweightness"[All Fields] OR "overweights"[All Fields]</td></tr>
<tr><td>smartphone: "smartphone"[MeSH Terms] OR "smartphone"[All Fields] OR "smartphones"[All Fields] OR "smartphone's"[All Fields]</td></tr>
<tr><td>smartwatch: "smartwatch"[All Fields] OR "smartwatches"[All Fields]</td></tr>
</table>
